# The Efficacy of Prophylactic Antibiotics on Post-Stroke Infections: An Updated Systematic Review and Meta-Analysis

**DOI:** 10.1038/srep36656

**Published:** 2016-11-14

**Authors:** Liang Liu, Xiao-Yi Xiong, Qin Zhang, Xiao-Tang Fan, Qing-Wu Yang

**Affiliations:** 1 Department of Neurology, Xinqiao Hospital, The Third Military Medical University, No. 183, Xinqiao Main street, Shapingba District, Chongqing 400037, China; 2 Department of Developmental Neuropsychology, School of Psychology, Third Military Medical University, Chongqing 400037, China

## Abstract

Post-stroke infections are common complications in acute stroke patients and are associated with an unfavorable functional outcome. However, reports on the effects of prophylactic antibiotics treatment on post-stroke infections are conflicting, especially those on post-stroke pneumonia and outcomes. We searched the PubMed, Embase, and Web of Knowledge databases up through March 11^th^, 2016. Seven randomized controlled trials including 4261 patients were analyzed among this systematic review and meta-analysis. We found preventive antibiotics treatment at the time of stroke onset did reduce the incidence of infections in adults with acute stroke (OR = 0.57, 95% CI: 0.38–0.85, *P* = 0.005), including reducing the number of urinary tract infections (OR = 0.34, 95% CI: 0.26–0.46, *P* < 0.001), but did not significantly decrease the rate of post-stroke pneumonia (OR = 0.91, 95% CI: 0.73–1.13, *P* = 0.385). Importantly, antibiotics treatment also showed no significant effect on the number of fatalities among stroke patients (OR = 1.07, 95% CI: 0.90–1.26, *P* = 0.743) and functional outcome scores on the modified Rankin Scale (OR = 1.76, 95% CI: 0.86–3.63, *p* = 0.124). Our study indicated that preventive antibiotics treatment not reduced the rate of post-stroke pneumonia or mortality, even though decreased the risk of infections, especially urinary tract infections. Thus, preventive antibiotics treatment may not be recommended for acute stroke patients.

Stroke is a leading cause of death worldwide. It is often followed by complications, especially infections, which occur in 30% of stroke patients and are strongly associated with unfavorable outcomes and high mortality^1^,^2^,^3^. Pneumonia and urinary tract infections were the most common types of post-stroke infections^2^. Furthermore, post-stroke pneumonia occurs in 10% of patients after an acute stroke, about half of which occur within the first 48 h after stroke onset^4^,^5^. Moreover, post-stroke pneumonia is associated with high mortality^6^. These results indicate that preventive anti-infection strategies may be beneficial for patients who have suffered a stroke.

Previous studies on the preventive use of antimicrobials in patients with acute stroke found conflicting results. Recently, a meta-analysis including 506 patients concluded that prophylactic antibiotics treatment after the onset of stroke significantly reduced the infection rate without major adverse effects, but had no effect on the mortality rate. Moreover, its effectiveness in reducing post-stroke pneumonia was ambiguous^7^. Further, existing guidelines for management of acute stroke state that prophylactic use of antibiotics is not recommended because it has not been proven to be effective^8^. Since the previous meta-analysis, there have been more multicenter randomized controlled trials with larger samples. Therefore, we performed this updated meta-analysis to systematically assess the effects of prophylactic antibiotics treatment on post-stroke infections and the occurrence of mortality among adult acute stroke patients. Importantly, we first take a subgroup meta-analysis of the effects of the preventive use of antimicrobials on the rate of post-stroke pneumonia and urinary tract infections.

## Results

### Characteristics of eligible studies

**Figure 1:**
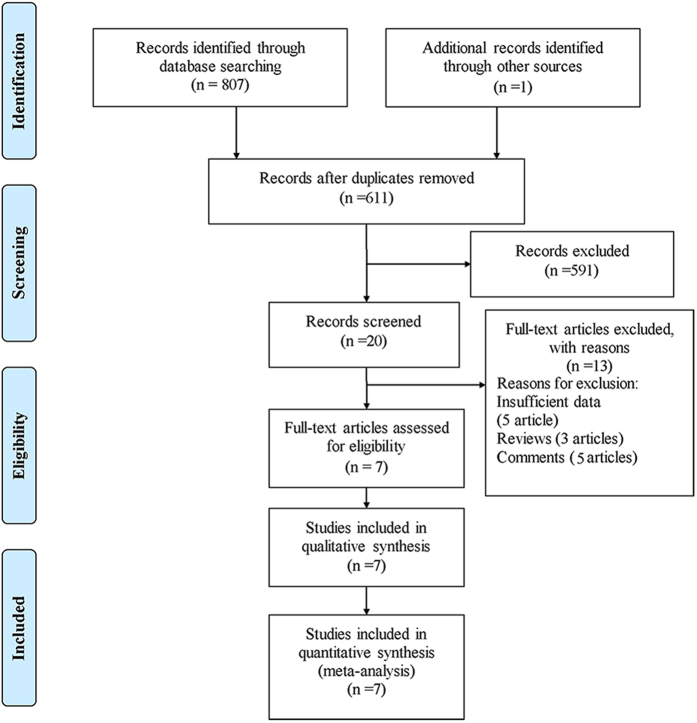
Flow diagram of study identification.

There were 808 records identified using different search strategies in three databases (345 from PubMed, 192 from Embase, and 270 from Web of Knowledge) and 1 record identified by ref. 9. After removing 197 duplications and 591 unrelated records by reading the titles and abstracts, 20 records warranted further screening through full-text reading. After full-text reading, 13 records were excluded including 5 records with insufficient data, 5 comments and 3 reviews. Finally, 7 studies fulfilled the eligibility criteria for inclusion in the primary analysis^9^,^10^,^11^,^12^,^13^,^14^,^15^. The study selection diagram is shown in Fig. 1.

The characteristics of the studies included in analysis are listed in Table 1. Those studies were published during 1998–2015. Four studies only included ischemic stroke patients^9^,^11^,^12^,^13^ and three studies included ischemic and hemorrhagic stroke patients^10^,^14^,^15^. Six studies were included for infection analysis. There were four studies with sufficient data for analyzing cases of pneumonia and urinary tract infections. The remaining six studies were involved in the analysis of mortality rates. The results of the Jadad Scale showed that four studies had high quality scores and three studies had low quality scores. A risk of bias table for each study is provided in Supplementary Table S1.

### Preventive Antibiotics Treatment at Stroke Onset and Outcomes

**Figure 2:**
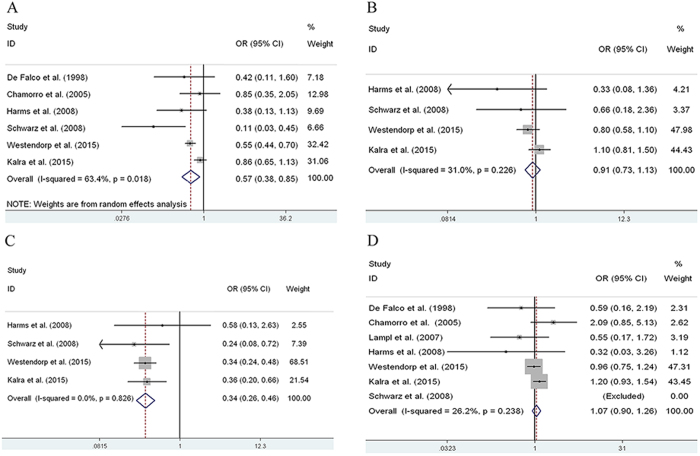
Forest plots of infections **(A),** pneumonia **(B),** urinary tract infections (**C**) and mortality **(D)** with prophylactic antibiotics treatment at stroke onset in observational studies. OR, odds ratio; CI, confidence interval.

Among the seven observational studies, 4261 patients were included. There were six studies used to analyze the effects of preventive antibiotics treatment on post-stroke infections, and one study did not provide efficiency data^11^. And the definitions used for infection in all included studies were presented in Supplementary Table S3. In all, 3831 patients (treatment group: 1926 patients; control group: 1905 patients) were included. The results of our meta-analysis suggested that preventive antibiotics treatment at the time of stroke onset reduced the occurrence of infections in adults with acute stroke (OR = 0.57, 95% CI: 0.38–0.85, *P* = 0.005) (Fig. 2A, Table 3).

However, among the subgroup analysis, only four studies were used to analyze the effects of preventive antibiotics treatment on post-stroke pneumonia, urinary tract infections and other infections, as the other studies did not report the occurrence rates. Thus, 3894 patients (treatment group: 1952 patients; control group: 1942 patients) were included. In this meta-analysis, we found that prophylactic antibiotics did not reduce the incidence of pneumonia in adult patients with acute stroke (OR = 0.91, 95% CI: 0.73–1.13, *P* = 0.385) (Fig. 2B, Table 3) and other infections (OR = 1.00, 95% CI: 0.63–1.60, *P* = 0.996) (Supplementary Figure S1), but decreased the incidence of urinary tract infections significantly (OR = 0.34, 95% CI: 0.26–0.46, *P* < 0.001) (Fig. 2C, Table 3).

**Figure 3:**
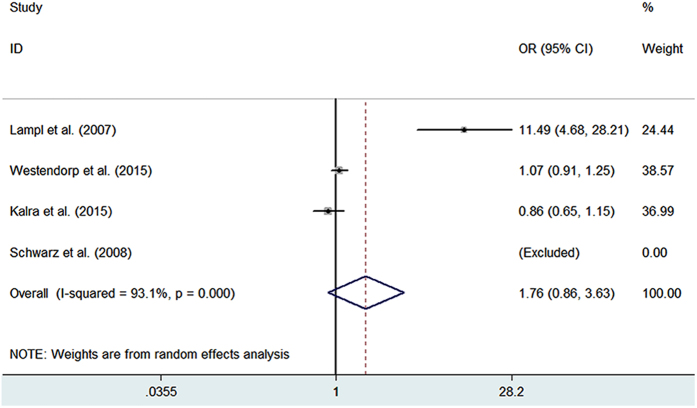
Forest plots of mRS (0–2) with prophylactic antibiotics treatment at stroke onset in observational studies. OR, odds ratio; CI, confidence interval.

Preventative antibiotics treatment at the time of stroke onset also did not reduce the mortality rate in adults with acute stroke (OR = 0.91, 95% CI: 0.73–1.13, *P* = 0.743) (Fig. 2D, Table 3). All of the studies included in this meta-analysis reported the mortality rate, but one study had no deaths in either the treatment group or control group^13^, so it was excluded, and the remaining studies including 4201 patients were analyzed (treatment group: 2101 patients; control group: 2100 patients). Indeed, as the modified Rankin Scale (mRS) score 0–2 was defined as a good outcome, preventative antibiotics treatment also did not show a benefit on the functional outcome evaluating by mRS score 0–2 (OR = 1.76, 95% CI: 0.86–3.63, *p* = 0.124) (Fig. 3 and Table 3). Besides, the antibiotic related side effects the outcome of neurological recovery were listed in Table 2.

### Heterogeneity analysis

Obvious between-study heterogeneity was found while estimating the effects of preventive antibiotics treatment on post-stroke infections (*I*^*2*^ = 63.4%, *P* = 0.018) (Fig. 2A, Table 3). To explore the between-study heterogeneity, a Galbraith plot was generated to graphically evaluate the sources. One study^13^ was outside the bounds of the Galbraith plot (Supplementary Figure S2), which was identified as the primary source of our between-study heterogeneity. Once this study was removed, the heterogeneity effectively decreased (*I*^*2*^ = 46.6%, *P* = 0.112, Table 3). Although no obvious heterogeneity was detected again among the remaining studies, the corresponding pooled OR changed little (OR = 0.65, 95% CI: 0.55–0.77, *P* < 0.001) (Supplementary Figure S3). Therefore, preventive antibiotics treatment at the time of stroke onset may significantly reduce the risk of infections in adults with acute stroke.

In contrast, among the occurrence rate of post-pneumonia, urinary tract infections and mortality analysis, there was no obvious between-study heterogeneity (*I*^*2*^ = 31.0%, *P* = 0.225; *I*^*2*^ = 0.0%, *P* = 0.826; *I*^*2*^ = 26.2%, *P* = 0.238, respectively) (Fig. 2B–D).

### Sensitivity analysis and publication bias

Each study was removed sequentially to verify the effect of each individual study on our on our results. No obvious changes were found after excluding any study. Therefore, our results were reliable (data not shown). Both Egger’s and Begg’s methods were applied to explore the publication bias in our meta-analysis. There was no significant publication bias among the occurrence rate of post-stroke infections, post-stroke pneumonia, urinary tract infections, mortality analysis in our study (Begg’s test: *P* = 0.348, *P* = 0.734, *P* = 0.497, *P* = 1.000 respectively). The funnel plots are shown in Supplementary Figure S5.

## Discussion

In this meta-analysis including data from 4261 patients, significant associations were found between preventive antibiotics treatment at stroke onset and post-stroke infections. We also found that the use of preventive antibiotics was not associated with a significant reduction in mortality rate. Compared with previous meta-analyses^7^,^16^, this meta-analysis contained more studies and the sample size was more than 8 times larger. Further, among our subgroups analysis we first found that preventive antibiotics treatment could not decrease the occurrence rate of post-stroke pneumonia, even though showed an effect on reducing urinary tract infections. We have found that no major side-effects were reported, although 2 patients developed exanthema or elevated liver enzymes with mezlocillin plus sulbactam treatment^13^.

Post-stroke infections were the most common complications of the acute phase after stroke, and most infections occurred within three days of hospital admission^17^. 30% of post-stroke patients experienced subsequent infection, including pneumonia and urinary tract infections that each occured in 10% of patients^2^. As the studies have been performed in general wards as well as ICU’s, pneumonia was mostly diagnosed within the first day following a stroke^18^,^19^,^20^. However, we found prophylactic antibiotic treatment did not decrease the occurrence rate of post-stroke pneumonia, although it was associated with a significant reduction in post-stroke infections and urinary tract infections. The most likely explanation is that post-stroke pneumonia may be a respiratory syndrome resulting from multiple factors, including medical complications and complex bacterial, chemical, and immunological causes that might not be preventable with antibiotics alone^14^,^21^. For example, acute stroke may lead to stroke-induced immunodepression by the cholinergic pathway, reducing lymphocyte count and delaying the recovery of T-lymphocyte loss, which may promote stroke patients developing post-stroke pneumonia^22^,^23^,^24^. More importantly, other existing preventive measures such as positioning, regular suction, swallowing techniques and modified diets may also be essential for patients with suspected post-stroke pneumonia in specialist stroke units^25^. For example, dysphagia screening, malnutrition, recumbency and tube feeding were associated with high risks for stroke patients developing pneumonia^26^,^27^. However, some studies found that a standardized multidisciplinary protocolized feeding tube placement has a beneficial role in preventing aspiration pneumonia in mildly or moderately disabled post-stroke patients^28^,^29^. Those may indicate that the stroke patient’s clinical conditions have a close link with pneumonia. Previous studies also found that patients with a higher stroke severity or lower levels of consciousness showed higher infection rates, in particular for pneumonia^30^,^31^. Further, the lesion location of a stroke also influenced the occurrence rate of post-stroke pneumonia. An ischemic lesion occurring in the anterior middle cerebral artery cortex has a higher susceptibility to stroke-associated infections^32^,^33^. Therefore, more normative cohort studies may elucidate why prophylactic antibiotics treatment could decrease the occurrence rate of post-stroke infections but have no effect on pneumonia.

In agreement with previous studies^7^,^16^, we also found that preventive antibiotics treatment at stroke onset could reduce the occurrence rate of post-stroke infections, which were associated with decreased functional outcome. Thus, the question arises as to why preventive antibiotics treatment did have a significant effect on post-stroke infections but no influence on the mortality rate. The explanation may be that infections could be just a marker or bystander of poor functional outcome, and preventive antibiotics treatment might not change the course of disease. On the other hand, as the modified Rankin Scale (mRS) score 0–2 was defined as a good outcome, out of four studies that reported the effects of preventive antibiotics treatment with a mRS score, one study had invalid data^13^. We also found that preventive antibiotics treatment showed no significant association with functional outcome. Thus, the other explanation may be that the type and dose of antibiotics were varied among the different studies. Indeed, some antibiotics showed neuroprotective properties in animal models of stroke, including minocycline and ceftriaxone^34^,^35^. Among ischemic stroke patients who received oral minocycline for 5 days, male but not female patients had significantly better neurological outcomes on day 90 than male patients^34^. Thus, in further studies, the male and female patients should be analyzed separately.

During analysis of the association between preventive antibiotics treatment and post-stroke infections, obvious heterogeneity was found. We noted that one study contributed to the heterogeneity across all included studies, which potentially confounded the analyses^13^. When we removed this study, the corresponding pooled RR value with very few changes was stable and reliable. The reason may be that this study excluded patients with a life expectancy of fewer than 90 days, possibly leaving only the less severely affected patients and, thus, overestimating the effect of preventive antibiotic therapy. Importantly, previous studies have confirmed that stroke severity was a risk factor for post-stroke infection^36^. Moreover, some potential bias had an important influence on the analysis, including publication bias and selection bias, but little evidence of publication bias was observed. Furthermore, only eligible studies published in English were included in this meta-analysis, so additional research in other populations is warranted to support generalization of the findings.

We acknowledge that there were several limitations in our study. First, relatively few studies were included in the meta-analysis, which restrict the strength and quality of the evidence. Only two studies used a randomized and double-blind design^10^,^12^, and other studies would increase the risk of bias and then may diminished the reliability of our results. Second, the diagnosis standard of infections were different among the studies included. Third, the inclusion criteria of antibiotic types were various among all included studies. It is possible that different antibiotic type may have varied beneficial effect against post-stroke infection. However, what a pity, all included studies did not present available data to conduct subgroup analysis by these factors (sex, stroke severity, and antibiotics type). Besides, even though we found some benefit of prophylactic antibiotic in urinary tract infection, we can’t identify what subgroup (e.g high NIHSS or elderly or antibiotic class) is better because those studies did not conduct subgroup analysis on urinary tract infection. The information regarding the cause of infections and death was unavailable, as was any individual patient data that might be used to establish the influence of different antibiotics treatments, dose–response relationships, or associations between sex and outcome. Lastly, there are no persuasive evidences to confirm whether antibiotics prophylaxis is good for stroke patients or not. Thus, larger randomized controlled trials are required.

## Methods

This meta-analysis and systematic review protocol has been follow the PRISMA (Preferred Reporting Items for Systematic Reviews and Meta-Analyses) statement.

### Search strategy and selection criteria

All published articles were searched in the PubMed, Embase, and Web of Knowledge databases up through March 11^th^, 2016. Key words were identified as follows: “stroke”, “cerebral infarction”, “brain infarction”, “cerebrovascular disease”, “cerebral hemorrhage”, “intracranial hemorrhage” or “cerebrovascular disorder”; and “infection”, “inflammation”, “fever”, “bacteraemia”, “sepsis”, or “pneumonia”; and “antimicrobial agents”, “antibiotics” or “prophylactic antibiotics”; and “prophylaxis” or “prevention”. The updated literature search, data extraction, and quality assessments were conducted independently by two authors (L.L., X.-Y. X.). All articles were retrieved and their references were checked to avoid missing other relevant articles. If data were not included in the original articles, we contacted the authors to obtain them.

### Inclusion and Exclusion Criteria

The inclusion criteria were as follows: (1) studies evaluating the effects of preventive antibiotics treatment in adult acute stroke patients (predefined as age 16 years or older); (2) randomized controlled trials were eligible for inclusion; and (3) The effect estimates of studies could be extracted or calculated from the available data. The exclusion criteria were as follows: (1) studies with insufficient data to calculate or extract effect estimates and (2) case reports, reviews, comments, abstracts or animal studies.

### Data Extraction

Two independent researchers (L.L., X.-Y. X.) conducted data extraction to ensure the reliability of the results. Disagreement was resolved by discussion. The information of studies meeting the inclusion criteria were extracted using a standardized extraction form. Relevant information included the first author’s name, publication year, inclusion criteria, exclusion criteria, intervention, outcomes, the numbers of cases and controls, the infection rate, urinary tract infections and pneumonia rate, other infections rate, the rate of mortality, mRS score, antibiotic related side effects and the Jadad Score.

### Assessment of Study Quality and Statistical Analysis

The quality of the studies was evaluated by two independent reviewers (L.L., X.Y.X.) according to the Jadad Scale, which contains three dimensions: randomization, blinding and withdrawals for case-control studies. The Jadad Scale is a validated 5-point scale assessing the following criteria: (1) whether randomized (yes = 1 point, no = 0); (2) whether randomization was described appropriately (yes = 1 point, no = 0); (3) whether double-blind (yes = 1 point, no = 0); (4) whether the double-blinding was described appropriately (yes = 1 point, no = 0); and (5) whether the dropouts and withdrawals were described (yes = 1 point, no = 0). The quality score ranges from 0 to 5 points, where 0–2 points is defined as a low-quality report study and a high quality study score is at least 3 points.

For all included studies, we (L.L and X.Y.X.) independently assessed the risk of bias for the following items: adequacy of sequence generation; allocation concealment; blinding of participants and personnel; blinding of outcome assessment; Incomplete outcome data; selective reporting; and other sources of bias. We have categorized these judgments as ’low’, ’high’, or ’unclear’ risk of bias^37^.

For each study, unadjusted odds ratios (ORs) and their corresponding 95% confidence intervals (CIs) were derived from patient numbers with each outcome categorized by antibiotics treatment. A Z test was conducted to assess the statistical significance of pooled ORs and 95% Cis. I-squared (I^2^) statistic and Chi-square-based Q-tests were used to investigate the heterogeneity across studies^38^. The selection of effects models according to our heterogeneity test was as follows: *P* > 0.10 for the Q test and I^2^ values less than 50% suggested no obvious heterogeneity across studies and a fixed (Mantel-Haenszel) effects model was applied; otherwise, a random (DerSimonian-Laird) effects model was used^39^. In addition, Galbraith plots were used to further explore the source of between-study heterogeneity. We also performed sensitivity analysis by removing each included study in sequence to assess the stability of our results. Publication bias was evaluated via a funnel plot using both Egger’s^40^ and Begg’s^41^ methods. Statistical significance was defined as a P-value <0.05. All statistical analyses were performed using STATA 12.0 software (Stata Corp, College Station, TX, USA).

## Additional Information

**How to cite this article**: Liu, L. *et al*. The Efficacy of Prophylactic Antibiotics on Post-Stroke Infections: An Updated Systematic Review and Meta-Analysis. *Sci. Rep.* **6**, 36656; doi: 10.1038/srep36656 (2016).

**Publisher’s note:** Springer Nature remains neutral with regard to jurisdictional claims in published maps and institutional affiliations.

## Supplementary Material

Supplementary Information

## Figures and Tables

**Table 1:** Characteristics of the studies included in the meta-analysis.

Author (publication year)	Inclusion criteria	Exclusion Criteria	Intervention	Outcomes	Inclusion NIHSS Treatment vs Control	Sample size	Infections Treatment vs Control	Pneumonia Treatment vs Control	Urinary tract infections Treatment vs Control	Mortality Treatment vs Control	Jadad Score
De Falco *et al*.^9^	Ischemic stroke within 12 hours	NA	Penicillin intramuscularly	Infectious complications, case fatality, functional outcome (BI, CNS)	CNS score: Mean (SD), 4.5 (2.3) vs 4.1 (2.1)	38/42	4/30 vs 8/30	NA	NA	4/38 vs 7/42	2
Chamorro *et al*.^10^	Stroke within 24 hours; age >18 y; NIHSS >score 4	Infection <3 months; T >37. 7 °C; allergy to fluoroquinolones; epilepsy; seizures; serum creatinine >2.5 mg/dL, antibiotics user; immunosuppressants therapy <3 months	Intravenous 500 mg/100 mL levofloxacin for 3 days, started within 24 h of stroke onset	Early infection (within 7 days), mortality, favorable outcome on day 90 (mRS <2, NIHSS <2, BI 95 or 100)	Median (interquartile), 14 (7–19) vs 11 (7–18)	67/69	11/67 vs 13/69	NA	NA	16/67 vs 9/69	5
Lampl *et al*.^11^	Ischemic stroke within 6 to 24 hours; age >18 y; NIHSS score >5	Hemorrhagic stroke; other disease; pre-existing neurologic disability; tetracycline allergic; renal failure; pre-existing infectious disease; swallowing difficulties	Orally minocycline 200 mg/d for 5 days, started within 6–24 h of stroke onset	NIHSS on day 90; NIHSS, mRS, BI, death on day 7, 30, 90	Mean (SD), 7.6 (3.8) vs 7.5 (3.2)	74/77	NA	NA	NA	5/74 vs 9/77	2
Harms *et al*.^12^	Ischemic stroke within 9 to 36 hours; aged ≥18 y; NIHSS score ≥11 in MCA territory	Hemorrhagic stroke; infections; antibiotics therapy <24 h; contraindications against moxifloxacin; immunosuppressant treatment	Intravenous 400 mg/d moxifloxacin for 5d, started within 36 h of stroke of onset	Infection rate on 11th day, bacterial spectrum, moxifloxacin resistance, body temperature, CRP, survival and functional outcome (BI) on day 180	Median (interquartile), 17 (12–21) vs 15 (12–25)	39/40	6/39 vs 13/40	3/39 vs 8/40	3/39 vs 5/40	1/39 vs 3/40	5
Schwarz *et al*.^13^	Ischemic stroke within 24 hours; age >18 y; NIHSS score >5	Hemorrhagic stroke; infections; renal insufficiency; penicillin or sulbactam allergic; immunosuppressant treatment; pregnancy	Intravenous mezlocillin 6 g/d plus sulbactam 1 g/d for 4 d, started within 24 h of stroke onset	mRS on day 90, infection, daily temperature	Median (interquartile), 17 (8–28) vs 15 (5–27)	30/30	15/30 vs 27/30	5/30 vs 7/30	8/30 vs 18/30	0/30 vs 0/30	2
Westendorp *et al*.^14^	Stroke within 24 hours; aged ≥18 y; NIHSS score ≥1	Infections; antibiotics therapy <24 h; pregnancy; penicillin or cephalosporins allergic; subarachnoid hemorrhage;	Intravenous 2 g/d ceftriaxone for 4d, started within 24 h of stroke of onset	mRS on 3 months, mortality, infection	Median (interquartile), 5 (3–9) vs 5 (3–9)	1268/1270	130/1268 vs 218/1270	71/1268 vs 88/1270	46/1268 vs 127/1270	131/1268 vs 136/1270	3
Kalra *et al*.^15^	Stroke within 48 hours and with dysphagia; aged >18 y	Allergic to antibiotics; infections; preexisting dysphagia; pyrexia; pregnancy; imminent death	amoxicillin or co-amoxiclav, plus clarithromycin for 7 d, started within 24 h of stroke of onset	Post-stroke pneumonia and mortality on day 14 and 90, mRS <2 on day 90, adverse events	Median (interquartile), 15 (9–20) vs 14 (9–20)	615/602	123/615 vs 136/602	101/615 vs 91/602	15/615 vs 39/602	184/615 vs 158/602	4

NA, not available; BI, Barthel Index; CNS, central nervous system; SD, standard deviation; NIHSS, National Institute of Health Stroke Scale.

T, temperature; mRS, modified Rankin Score; MCA, middle cerebral artery; CRP, C-reactive protein.

**Table 2:** The outcome of antibiotic related side effects and the outcome of neurological recovery.

**Author (publication year)**	**Diarrhea Treatment vs Control**	**Multi-drug resistant infection Treatment vs Control**	**mRS score 0–2 Treatment vs Control**
De Falco *et al*.^9^	NA	NA	NA
Chamorro *et al*.^10^	NA	NA	NA
Lampl *et al*.^11^	NA	NA	67/74 vs 35/77
Harms *et al*.^12^	2/40 vs 2/39	NA	NA
Schwarz *et al*.^13^	NA	NA	0/30 vs 0/ 30
Westendorp *et al*.^14^	2/1242 vs 0/1270	6/1242 vs 5/1270	781/1268 vs 763/1270
Kalra *et al*.^15^	2/615 vs 4/602	11/615 vs 14/602	109/595 vs 121/586

NA, not available.

**Table 3:** Summary of meta-analysis results.

Groups	Studies	Test of association				Heterogeneity		
		OR[95%CI]	p value	Model	Z	Χ^2^	p value	*I*^2^(%)
Infections	6	0.57[0.39–0.82]	0.003	RE	3	11.77	0.038	57.50%
Infections (except one study)	5	0.63[0.53–0.75]	<0.001	FE	5.18	5.81	0.214	31.1%,
Pneumonia	4	0.91[0.73–1.13]	0.385	FE	0.87	4.35	0.225	31.0%,
Urinary tract infections	4	0.34[0.26–0.46]	<0.001	FE	7.37	0.9	0.826	0.00%
mRS (0–2)	4	1.76[0.86–3.63]	0.124	RE	1.54	29.08	<0.001	93.1%

RE, random effects; FE, fixed effects; OR, odds ratio; CI, confidence interval.

## References

[b1] Vermeij F. H. . Stroke-associated infection is an independent risk factor for poor outcome after acute ischemic stroke: data from the Netherlands Stroke Survey. Cerebrovasc Dis 27, 465–471 (2009). 19329851 10.1159/000210093

[b2] Westendorp W. F., Nederkoorn P. J., Vermeij J. D., Dijkgraaf M. G. & van de Beek D. Post-stroke infection: a systematic review and meta-analysis. BMC neurology 11, 110 (2011). 21933425 10.1186/1471-2377-11-110 PMC3185266

[b3] Popovic N. . The frequency of poststroke infections and their impact on early stroke outcome. Journal of stroke and cerebrovascular diseases: the official journal of National Stroke Association 22, 424–429 (2013). 23540255 10.1016/j.jstrokecerebrovasdis.2013.03.003

[b4] Finlayson O. . Risk factors, inpatient care, and outcomes of pneumonia after ischemic stroke. Neurology 77, 1338–1345 (2011). 21940613 10.1212/WNL.0b013e31823152b1

[b5] Emsley H. C. & Hopkins S. J. Acute ischaemic stroke and infection: recent and emerging concepts. The Lancet. Neurology 7, 341–353 (2008). 18339349 10.1016/S1474-4422(08)70061-9

[b6] Katzan I. L., Cebul R. D., Husak S. H., Dawson N. V. & Baker D. W. The effect of pneumonia on mortality among patients hospitalized for acute stroke. Neurology 60, 620–625 (2003). 12601102 10.1212/01.wnl.0000046586.38284.60

[b7] Westendorp W. F. . Antibiotic therapy for preventing infections in patients with acute stroke. The Cochrane database of systematic reviews 1, Cd008530 (2012). 22258987 10.1002/14651858.CD008530.pub2

[b8] Adams H. P. Jr. . Guidelines for the early management of adults with ischemic stroke: a guideline from the American Heart Association/American Stroke Association Stroke Council, Clinical Cardiology Council, Cardiovascular Radiology and Intervention Council, and the Atherosclerotic Peripheral Vascular Disease and Quality of Care Outcomes in Research Interdisciplinary Working Groups: The American Academy of Neurology affirms the value of this guideline as an educational tool for neurologists. Circulation 115, e478–e534 (2007). 17515473 10.1161/CIRCULATIONAHA.107.181486

[b9] De Falco F. A., Santangelo R., Majello L. & Angelone P. Antimicrobial prophylaxis in the management of ischemic stroke. Rivista di Neurobiologia 44, 63–67 (1998).

[b10] Chamorro A. . The Early Systemic Prophylaxis of Infection After Stroke study: a randomized clinical trial. Stroke; a journal of cerebral circulation 36, 1495–1500 (2005). 15961713 10.1161/01.STR.0000170644.15504.49

[b11] Lampl Y. . Minocycline treatment in acute stroke: an open-label, evaluator-blinded study. Neurology 69, 1404–1410 (2007). 17909152 10.1212/01.wnl.0000277487.04281.db

[b12] Harms H. . Preventive antibacterial therapy in acute ischemic stroke: a randomized controlled trial. PloS one 3, e2158 (2008). 18478129 10.1371/journal.pone.0002158 PMC2373885

[b13] Schwarz S., Al-Shajlawi F., Sick C., Meairs S. & Hennerici M. G. Effects of prophylactic antibiotic therapy with Mezlocillin plus sulbactam on the incidence and height of fever after severe acute ischemic stroke: The Mannheim infection in stroke study (MISS). Stroke; a journal of cerebral circulation 39, 1220–1227 (2008). 18309164 10.1161/STROKEAHA.107.499533

[b14] Westendorp W. F. . The Preventive Antibiotics in Stroke Study (PASS): a pragmatic randomised open-label masked endpoint clinical trial. Lancet (London, England) 385, 1519–1526 (2015). 10.1016/S0140-6736(14)62456-9 25612858

[b15] Kalra L. . Prophylactic antibiotics after acute stroke for reducing pneumonia in patients with dysphagia (STROKE-INF): a prospective, cluster-randomised, open-label, masked endpoint, controlled clinical trial. Lancet (London, England) 386, 1835–1844 (2015). 10.1016/S0140-6736(15)00126-9 26343840

[b16] van de Beek D. . Preventive antibiotics for infections in acute stroke: a systematic review and meta-analysis. Archives of neurology 66, 1076–1081 (2009). 19752296 10.1001/archneurol.2009.176

[b17] Ionita C. C. . Acute ischemic stroke and infections. Journal of stroke and cerebrovascular diseases: the official journal of National Stroke Association 20, 1–9 (2011). 20538486 10.1016/j.jstrokecerebrovasdis.2009.09.011

[b18] Hilker R. . Nosocomial pneumonia after acute stroke: implications for neurological intensive care medicine. Stroke; a journal of cerebral circulation 34, 975–981 (2003). 12637700 10.1161/01.STR.0000063373.70993.CD

[b19] Davenport R. J., Dennis M. S., Wellwood I. & Warlow C. P. Complications after acute stroke. Stroke; a journal of cerebral circulation 27, 415–420 (1996). 8610305 10.1161/01.str.27.3.415

[b20] Nakajima M., Watanabe-Hara R., Inatomi Y., Hashimoto Y. & Uchino M. Respiratory infectious complications after acute ischemic stroke. Rinsho shinkeigaku=Clinical neurology 42, 917–921 (2002). 12739378

[b21] Ji R. . Interrelationship among common medical complications after acute stroke: pneumonia plays an important role. Stroke; a journal of cerebral circulation 44, 3436–3444 (2013). 24178914 10.1161/STROKEAHA.113.001931

[b22] Haeusler K. G. . Cellular immunodepression preceding infectious complications after acute ischemic stroke in humans. Cerebrovasc Dis 25, 50–58 (2008). 18033958 10.1159/000111499

[b23] Vogelgesang A. . Analysis of lymphocyte subsets in patients with stroke and their influence on infection after stroke. Stroke; a journal of cerebral circulation 39, 237–241 (2008). 18048864 10.1161/STROKEAHA.107.493635

[b24] Engel O. . Cholinergic Pathway Suppresses Pulmonary Innate Immunity Facilitating Pneumonia After Stroke. Stroke; a journal of cerebral circulation 46, 3232–3240 (2015). 26451017 10.1161/STROKEAHA.115.008989

[b25] Hoffmeister L. . Performance measures for in-hospital care of acute ischemic stroke in public hospitals in Chile. BMC neurology 13, 23 (2013). 23496941 10.1186/1471-2377-13-23 PMC3599613

[b26] Brogan E., Langdon C., Brookes K., Budgeon C. & Blacker D. Dysphagia and factors associated with respiratory infections in the first week post stroke. Neuroepidemiology 43, 140–144 (2014). 25402187 10.1159/000366423

[b27] Matsumura T., Mitani Y., Oki Y., Fujimoto Y. & Ishikawa A. Risk factors for the onset of aspiration pneumonia among stroke patients in the recovery stage. Nihon Ronen Igakkai zasshi. Japanese journal of geriatrics 51, 364–368 (2014). 25327371 10.3143/geriatrics.51.364

[b28] Nakajoh K. . Relation between incidence of pneumonia and protective reflexes in post-stroke patients with oral or tube feeding. Journal of internal medicine 247, 39–42 (2000). 10672129 10.1046/j.1365-2796.2000.00565.x

[b29] Gandolfi M. . Improving post-stroke dysphagia outcomes through a standardized and multidisciplinary protocol: an exploratory cohort study. Dysphagia 29, 704–712 (2014). 25115857 10.1007/s00455-014-9565-2

[b30] Hinchey J. A. . Formal dysphagia screening protocols prevent pneumonia. Stroke; a journal of cerebral circulation 36, 1972–1976 (2005). 16109909 10.1161/01.STR.0000177529.86868.8d

[b31] Kwon H. M., Jeong S. W., Lee S. H. & Yoon B. W. The pneumonia score: a simple grading scale for prediction of pneumonia after acute stroke. American journal of infection control 34, 64–68 (2006). 16490608 10.1016/j.ajic.2005.06.011

[b32] Harms H. . Influence of stroke localization on autonomic activation, immunodepression, and post-stroke infection. Cerebrovasc Dis 32, 552–560 (2011). 22104620 10.1159/000331922

[b33] Kodumuri N. . The association of insular stroke with lesion volume. NeuroImage. Clinical 11, 41–45 (2016). 26909326 10.1016/j.nicl.2016.01.007 PMC4732185

[b34] Amiri-Nikpour M. R., Nazarbaghi S., Hamdi-Holasou M. & Rezaei Y. An open-label evaluator-blinded clinical study of minocycline neuroprotection in ischemic stroke: gender-dependent effect. Acta Neurol Scand 131, 45–50 (2015). 25155474 10.1111/ane.12296

[b35] Lujia Y. . Ceftriaxone pretreatment protects rats against cerebral ischemic injury by attenuating microglial activation-induced IL-1beta expression. The International journal of neuroscience 124, 657–665 (2014). 24985046 10.3109/00207454.2013.856009

[b36] Bustamante A. . Ischemic stroke outcome: A review of the influence of post-stroke complications within the different scenarios of stroke care. European journal of internal medicine 29, 9–21 (2016). 26723523 10.1016/j.ejim.2015.11.030

[b37] Higgins J. P. T. Green S (editors). Cochrane Handbook for Systematic Reviews of Interventions Version 5.1.0 [updated March 2011]. The Cochrane Collaboration (2011). Available from www.cochrane-handbook.org.

[b38] Higgins J. P., Thompson S. G., Deeks J. J. & Altman D. G. Measuring inconsistency in meta-analyses. Bmj 327, 557–560 (2003). 12958120 10.1136/bmj.327.7414.557 PMC192859

[b39] Borenstein M. & Higgins J. P. Meta-analysis and subgroups. Prevention science: the official journal of the Society for Prevention Research 14, 134–143 (2013). 23479191 10.1007/s11121-013-0377-7

[b40] Egger M., Davey Smith G., Schneider M. & Minder C. Bias in meta-analysis detected by a simple, graphical test. Bmj 315, 629–634 (1997). 9310563 10.1136/bmj.315.7109.629 PMC2127453

[b41] Begg C. B. & Mazumdar M. Operating characteristics of a rank correlation test for publication bias. Biometrics 50, 1088–1101 (1994). 7786990

