# Supplementary material for: The Efficacy of Prophylactic Antibiotics on Post-Stroke Infections: An Updated Systematic Review and Meta-Analysis
Source: Sci Rep. 2016 Nov 14;6:36656. doi: 10.1038/srep36656 (PMC5107889; doi:10.1038/srep36656)
Supplement: Supplementary Information [file srep36656-s1.pdf]

# Supplementary information

**The Efficacy of Prophylactic Antibiotics on Post-Stroke Infections: An Updated Systematic Review and Meta-Analysis**


Liang Liu, MD[1*]; Xiao-Yi Xiong, PhD[1*]; Qin Zhang, MD[1]; Xiao-Tang Fan, PhD[2]; Qing-Wu Yang, MD[1]

[1]Department of Neurology, Xinqiao Hospital, The Third Military Medical University, No. 183, Xinqiao Main street, Shapingba District, Chongqing 400037, China

[2]Department of Developmental Neuropsychology, School of Psychology, Third Military Medical University, Chongqing 400037, China

[*] This authors contributed equally to this work.

**Correspondence to:**

Qing-Wu Yang, email: yangqwmlys@hotmail.com

Xiao-Tang Fan, email: fanxiaotang2005@163.com

**Supplemental Figures and Figure Legends:**

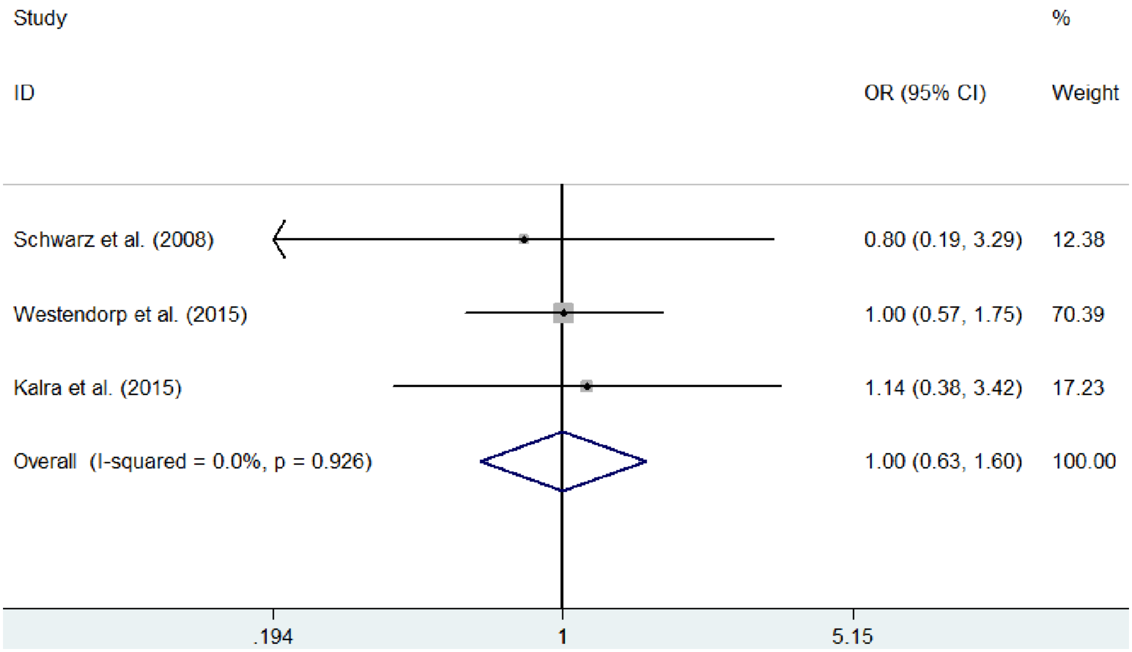


**Supplementary Figure S1.** Forest plots of other infections with prophylactic antibiotics treatment at stroke onset in observational studies. OR, odds ratio; CI, confidence interval.

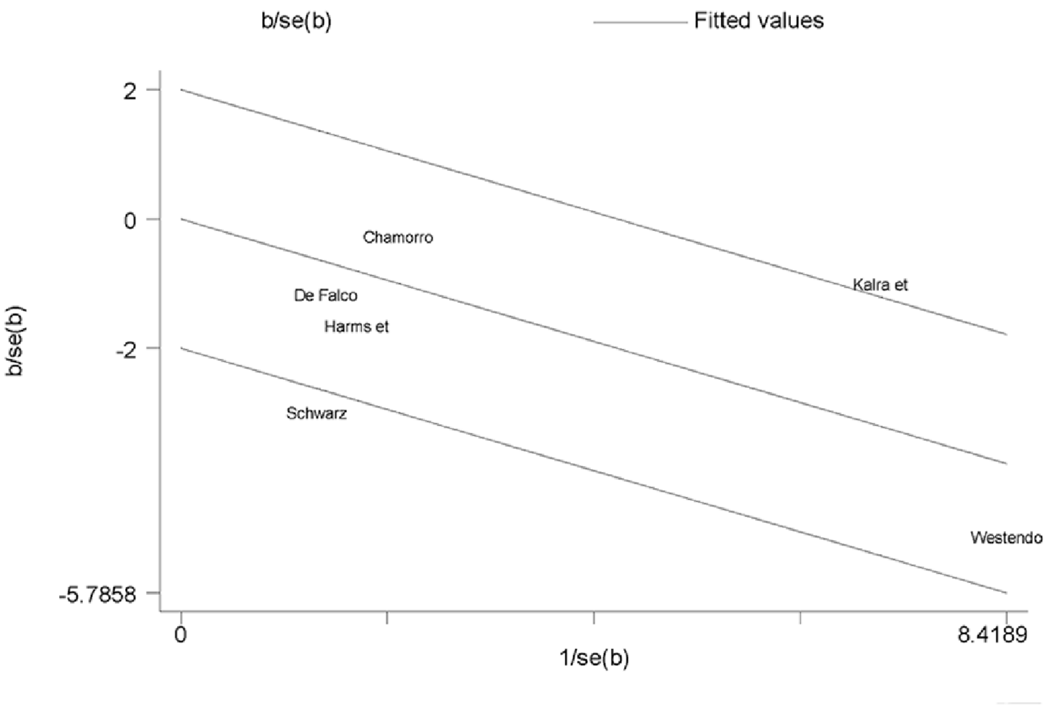


**Supplementary Figure S2.** Galbraith plot of prophylactic antibiotics treatment at stroke onset and the occurrence rate ofinfections.

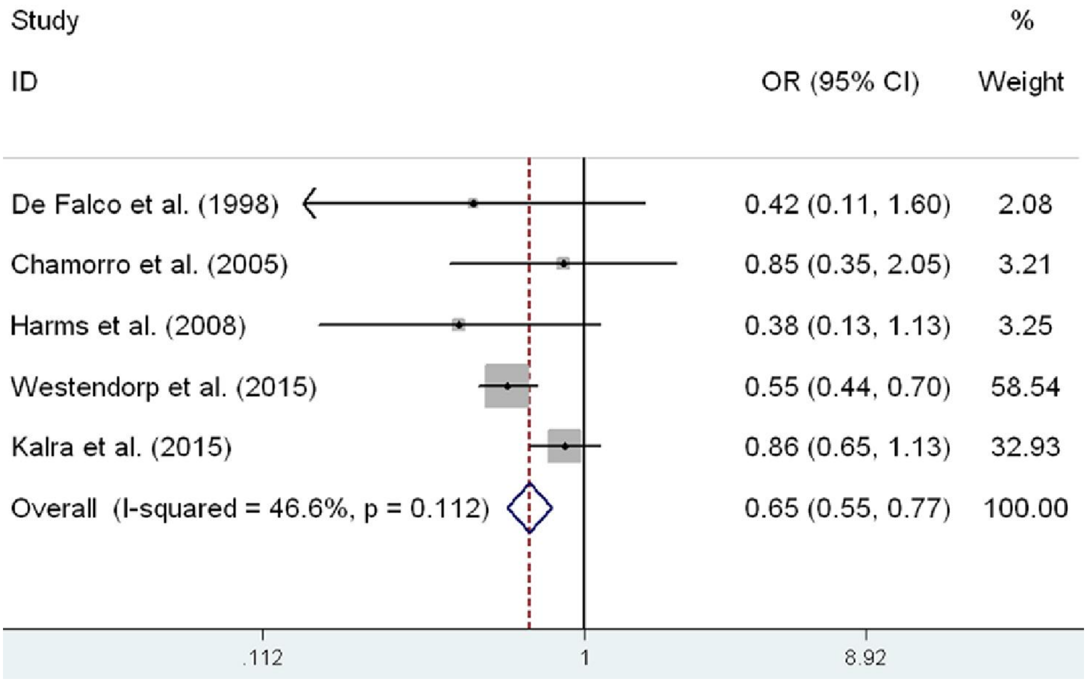


**Supplementary Figure S3.** Forest plot of prophylactic antibiotics treatment at stroke onset and the occurrence rate of infections after removing one study. OR, odds ratio; CI, confidence interval.

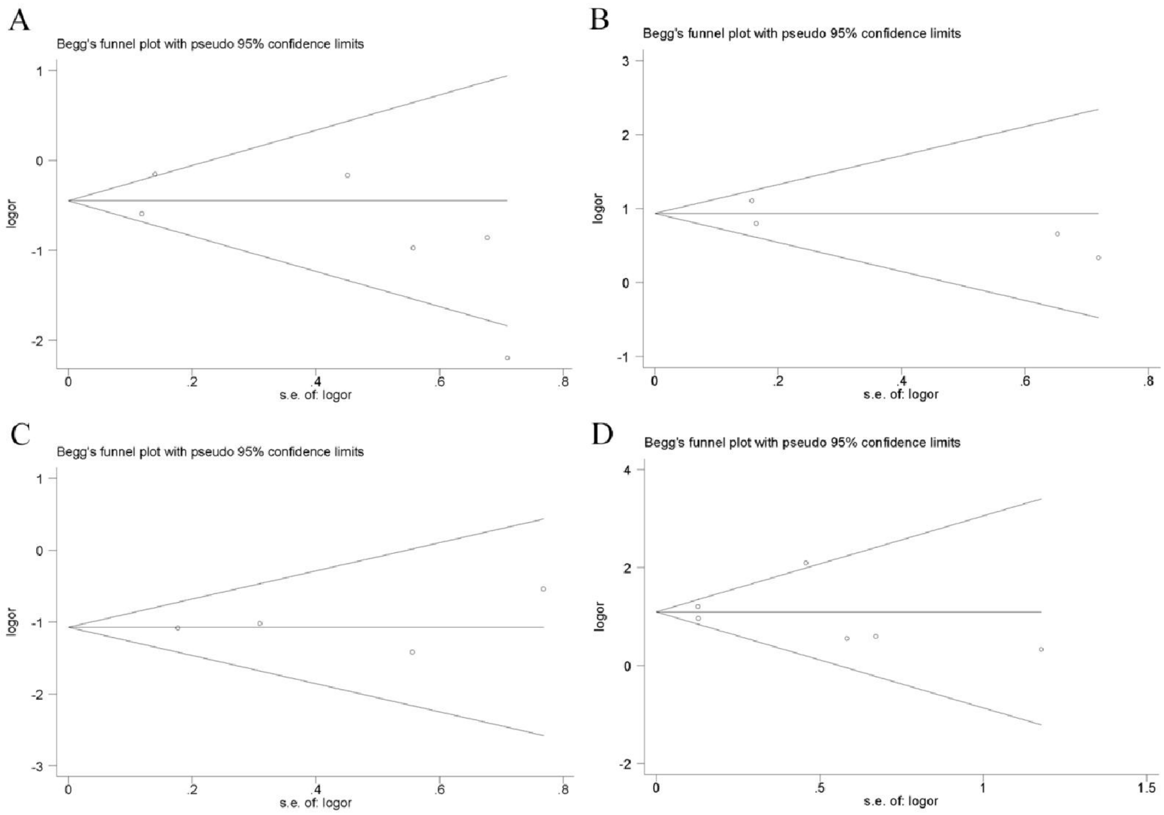


**Supplementary Figure S4.** Funnel plots of studies examining the association between prophylactic antibiotics treatment at stroke onset and the occurrence rate of infections **(A),** pneumonia **(B),** urinary tract infections **(C)** and mortality **(D).**

**Supplementary Table S1**. The risk of bias assessment of included studies

| Author (publication year) | Bias | Authors' judgement | Support for judgement |
|---|---|---|---|
| De Falco et al. (1998) | Random sequence generation (selection bias) | Low risk | This study is conducted randomly |
| | Allocation concealment (selection bias) | Unclear risk | This study not mention allocation concealment |
| | Blinding (performance bias and detection bias) All outcomes | High risk | Blinding of outcome assessment is not described |
| | Incomplete outcome data (attrition bias) All outcomes | High risk | No reports about completeness of follow-up and outcome assessment |
| | Selective reporting (reporting bias) | High risk | Outcome assessment did not perform at a fixed time point |
| | Other bias | Low risk | No found |

| | | | |
|---|---|---|---|
| Chamorro et al. (2005) | Random sequence generation (selection bias) | Low risk | This study is conducted randomly |
| | Allocation concealment (selection bias) | Low risk | Study treatment was prepared at the central pharmacy of the institution and kept within its premises until allocation |
| | Blinding (performance bias and detection bias) All outcomes | Low risk | This is a double-blind and placebo controlled study. All outcomes were evaluated blindly |
| | Incomplete outcome data (attrition bias) All outcomes | Low risk | There were no losses to follow-up |
| | Selective reporting (reporting bias) | Low risk | All outcomes are reported |
| | Other bias | Low risk | No found |

| | | | |
|---|---|---|---|
| Lampl et al. (2007) | Random sequence generation (selection bias) | Low risk | This study is conducted randomly by using the 8th number of the subject's identity card |
| | Allocation concealment (selection bias) | High risk | Physicians were aware of the treatment because they knew that patients with even/odd NID numbers would get a certain treatment |
| | Blinding (performance bias and detection bias) All outcomes | Low risk | Outcomes were assessed blindly |
| | Incomplete outcome data (attrition bias) All outcomes | High risk | Some outcomes (Scores on NIHSS, BI and mRS) are presented as means. The number of patients lost to follow-up is not mentioned. |
| | Selective reporting (reporting bias) | Low risk | All outcomes are reported |
| | Other bias | Low risk | No found |

| | | | |
|---|---|---|---|
| Harms et al. (2008) | Random sequence generation (selection bias) | Low risk | A computer generated allocation schedule was used |
| | Allocation concealment (selection bias) | Low risk | The necessary labelling, ensuring the blinding for patients, nursing personnel and investigating physicians shall be undertaken by the institute |
| | Blinding (performance bias and detection bias) All outcomes | Low risk | This is a double-blind and placebo controlled study. All outcomes were evaluated blindly |
| | Incomplete outcome data (attrition bias) All outcomes | High risk | There were 7 patients lost to follow-up |
| | Selective reporting (reporting bias) | Low risk | All outcomes are reported |
| | Other bias | Low risk | No found |

| | | | |
|---|---|---|---|
| Schwarz et al. (2008) | Random sequence generation (selection bias) | Low risk | A computer generated allocation schedule was used |
| | Allocation concealment (selection bias) | Low risk | Each number was hidden in a sealed envelope |
| | Blinding (performance bias and detection bias) All outcomes | High risk | The assessment of the score of NIHSS and mRS did not conduct blindly |
| | Incomplete outcome data (attrition bias) All outcomes | Low risk | There were no losses to follow-up |
| | Selective reporting (reporting bias) | Low risk | All outcomes are reported |
| | Other bias | Low risk | No found |

| | | | |
|---|---|---|---|
| Westendorp et al. (2015) | Random sequence generation (selection bias) | Low risk | Randomisation was done with an online tool |
| | Allocation concealment (selection bias) | High risk | The patient and the treating physician were aware of the treatment assignment |
| | Blinding (performance bias and detection bias) All outcomes | Low risk | The research nurses who did the follow-up interviews were masked to treatment allocation |
| | Incomplete outcome data (attrition bias) All outcomes | High risk | There were 24 patients lost to follow-up |
| | Selective reporting (reporting bias) | Low risk | All outcomes are reported |
| | Other bias | Low risk | No found |

| | | | |
|---|---|---|---|
| Kalra et al. (2015) | Random sequence generation (selection bias) | Low risk | Randomisation was computer generated and done away from the trial office |
| | Allocation concealment (selection bias) | Low risk | Randomisation was computer generated and admitted directly to specialist care |
| | Blinding (performance bias and detection bias) All outcomes | Low risk | Patients, research staff obtaining data, and statisticians undertaking analyses of the outcome data were unaware of stroke unit allocation. |
| | Incomplete outcome data (attrition bias) All outcomes | High risk | 34 patients lost to follow-up |
| | Selective reporting (reporting bias) | Low risk | All outcomes are reported |
| | Other bias | Low risk | No found |

**Supplementary Table S2.** The data about other infections of the included studies.

| **Author (publication year)** | **Other Infections Type** | **Other Infections Treatment vs Control** |
|---|---|---|
| Schwarz et al. (2008) | Tracheobronchitis | 2/30 vs 3/30 |
| | Other/unclear origin | 2/30 vs 2/30 |
| Westendorp et al. (2015) | Other infections (unclear) | 25/1268 vs 25/1270 |
| Kalra et al. (2015) | Other infections (unclear) | 7/615 vs 6/602 |

**Supplementary Table S3**. Definitions Used for Infection

| Author (publication year) | Source of infection definition |
|---|---|
| De Falco et al. (1998) | NA |
| Chamorro et al. (2005) | Infection was defined if temperature>37.5°C in 2 determinations or>37.8°C in a single determination in patients with suggestive symptoms (ie, cough, dyspnea, pleuritic pain, urinary tract symptoms), white blood cell count >11 000/mL or>4000/mL, pulmonary infiltrate on chest x-rays, or cultures positive for a pathogen. Otherwise, temperature>37.8°C was classified as noninfectious hyperthermia. |
| Lampl et al. (2007) | NA |
| Harms et al. (2008) | Criteria modified from US Centers for Disease Control and Prevention criteria |
| Schwarz et al. (2008) | Criteria from Paul Ehrlich Society for chemotherapy |
| Westendorp et al. (2015) | First, clinical diagnosis according to the treating physician will be recorded. Second, diagnosis of infection the modified criteria of the United States Centres for Disease Control and Prevention |
| Kalra et al. (2015) | Criteria for pneumonia from the Centres for Disease Control and Prevention |

NA, not available.
